# Attention and Sentiment of the Chinese Public toward a 3D Greening System Based on Sina Weibo

**DOI:** 10.3390/ijerph20053972

**Published:** 2023-02-23

**Authors:** Wenlu Zhao, Guanghu Jin, Chenyue Huang, Jinji Zhang

**Affiliations:** 1 College of Agriculture, Yanbian University, Yanji 133002, China; 2 College of Engineering, Yanbian University, Yanji 133002, China

**Keywords:** three-dimensional greenery systems (TGS), the Chinese public, Sina Weibo, sentiment, web crawler, text mining

## Abstract

The rapid development of global urbanization over the years has led to a significant increase in the urban population, resulting in an imbalance in the urban green space structure. Transforming the urban 2D space green quantity into a 3D space green quantity to create 3D greenery systems (TGS) is a space resource that cannot be ignored in the process of urban green space expansion. This research gathered and analyzed Sina Weibo post information and user information related to TGS to investigate the changing trend of attention status and emotional orientation of the Chinese public on TGS. We employed web crawler technology and text mining to search and analyze the data on the Sina Weibo platform. This research aids policymakers and stakeholders in comprehending the general public’s perspective on TGS and showing the transmission channel of public sentiment and the origins of negative sentiment. Results indicate that the public’s attention to TGS has greatly increased since the shift in the government’s idea of governance, although it still needs improvement. Despite TGS’s good thermal insulation and air purification effects, 27.80% of the Chinese public has a negative attitude toward it. The public’s negative sentiment of TGS housing is not solely due to pricing. The public is mainly concerned about the damage to the structure of buildings caused by TGS, the subsequent maintenance of plants, the increase in indoor mosquitoes, and lighting and humidity problems. This research helps decision makers understand the public opinion communication process via social media and provides corresponding solutions, which is of great significance for the future development of TGS.

## 1. Introduction

With the rapid development of economy and society, environmental problems such as global warming, biodiversity reduction, noise increases, and pollution increases are becoming increasingly serious, and the global ecological environment is facing great challenges. Nature-based Solutions (NbS), implementation actions inspired and supported by nature, have recently emerged as one of the key strategies for achieving sustainable development across all environmental, economic, and social perspectives [1]. They aim to address a range of environmental challenges in an efficient and adaptable manner, while providing economic, social, and environmental benefits [1]. Over the years, the rapid development of the city has neglected the land’s ecological and environmental capacity; artificial impermeable materials have replaced native vegetation [2]; and the city has a large number of building roofs, walls, and hard floors [3]. This phenomenon has led to numerous environmental problems, most notably air pollution and the urban heat island (UHI) effect [4]. People all throughout the world want nature to return to cities and to develop livable and green homes. Due to the increasingly serious urbanization, the urban population continues to increase, which also makes the environmental benefits of urban green space more significant. Increasing the greenery density on the urban ground has gradually become a challenge (such as parks), and TGS has become an NbS with great potential to increase the greenery density. Rapid urbanization, restricted land resources, and insufficient vegetation are all motivations for developing TGS. TGS have important ecosystem functional services. Studies have shown that TGS has significant effects in improving the UHI effect [5,6], alleviating urban floods, reducing air pollution, and improving urban biodiversity [7].

Three-dimensional greenery systems (TGS) are a general term for various forms of greening built with buildings (structures) as the carrier and plant materials as the main body, mainly including green roofs, vertical greenery systems (VGS), greening along the mouth, and trellis greening [8]. TGS offers the advantages of less area, a fast landscape effect, and a high greening rate. Furthermore, TGS is not a novel idea, and its history dates back to the Babylonian period in Europe [9]. Different countries have taken varying actions to increase the TGS of buildings and reduce the negative effects of urbanization. In 1986, the Federal Building Act issued by Germany defined green roofs as one of the means of ecological compensation and source control [10]. In the United States, green roofs and wall greening are included in Leadership in Energy and Environmental Design (LEED) [11]. Singapore’s Skyrise Greenery Incentive Scheme 2.0 provides funding for up to 50% of the installation expenses of rooftops and vertical greenery in 2015 [12]. Tokyo, Japan, has made green roofs mandatory for new and expanded buildings larger than 1000 m^2^ [13]. The combination of architecture and greening and the realization of urban greening by using 3D space have become popular research topics.

TGS has gradually realized its globalization, scale, diversification, and legal development as a result of its continual development. China is the world’s most populous country. With increasing urbanization and industrialization, problems such as biodiversity loss, habitat fragmentation and environmental quality deterioration have emerged. TGS has become an NbS with great potential to increase the greenery density. Therefore, it is necessary to take China as an example to study TGS. In recent years, TGS exploration and research in China have mainly focused on roof gardens, VGS and other forms of greening, TGS technology, or the selection and cultivation of plants and soil. The report of the 20th National Congress of the Communist Party of China emphasizes green development and harmonious coexistence between man and nature [14]. These initiatives include speeding the green transformation of the development model, intensifying the prevention and control of environmental pollution, diversifying the ecosystem, and actively and consistently supporting peak carbon dioxide emissions and carbon neutrality [14]. However, despite the significant urban TGS demand, its actual construction is still limited. In this case, the public’s perception of TGS must be understood, and the reasons behind the slow development of TGS in China must be explored. Supporting public participation is considered an important aspect of China’s sustainable development strategy [2]. 

Few studies have employed objective large data sets to assess the public’s perspective toward TGS, while the majority of prior studies relied on questionnaires or interviews. The main 10 studies were as follows. Sarwar et al. [15] collected a structured questionnaire from 400 residents of Lahore, Pakistan, to explore their opinions, perceptions, and barriers to adopting green roof technology. Liberalesso et al. [16] collected 345 structured questionnaires from five hostels in Lisbon, Portugal, in order to understand respondents’ perceptions and preferences regarding green roofs and green walls. To evaluate the effectiveness of green roofs in Hong Kong, Tam et al. [17] collected 357 questionnaires from green roof-related professionals, users, and owners. The results show that respondents are very interested in green roofs but not willing to invest in green roofs. Hui et al. [18] collected 477 and 500 questionnaires from Hong Kong and Beijing, respectively, to compare public perceptions of green roofs and green walls across cities for the first time. Tsantopoulos et al. [19] conducted a questionnaire survey and interview with 800 apartment owners in Athens, Greece, in order to understand the views of residents in Athens on building TGS. The results show that most respondents are willing to install green roofs and vertical gardens, but they want government subsidies. To assess public perceptions of the living walls of the ‘Quironsalud Sagrado Corazon’ Hospital in Seville, Spain, Perez-Urrestarazu et al. [20] administered a questionnaire to 550 patients, staff, and visitors within the hospital. Most of the respondents said that a living wall played a positive role in improving people’s psychology and health. Wang et al. [21] conducted a questionnaire survey of companies and practitioners involved in Indoor vertical greenery system (iVGS) in Malaysia to determine their views on iVGS. A total of 169 questionnaires were collected, and the results showed that aesthetics was the most significant benefit of iVGS. Yuliani et al. [22] collected 591 surveys of Indonesian people’s views on green roofs. The results show that the public’s understanding of green roofs is very high, and the development of green roofs needs to be promoted according to the local culture. Alqahtany [23] collected 380 questionnaires on public perceptions of green roofs in Riyadh, Saudi Arabia. The results showed that 91% of respondents identified climate as the biggest challenge facing green roofs. Mesimaki et al. [24] surveyed 178 people in Helsinki, Finland, about their views and preferences on green roofs and collated their opinions. During sampling and analysis, questionnaires and interviews contain the researcher’s subjective judgment, which may help preserve consistency in low-sample studies [25]. In large-scale investigations, investigators cannot simply make subjective judgments on the same level, which might result in bias [25]. Using big data to investigate public attitudes can compensate for the lack of research in this area to a certain extent. It takes a lot of time and experience to collect questionnaires, but it is difficult to guarantee the recovery rate and quality of questionnaires. To fill this research gap, this study used an objective large data set to analyze public opinions on TGS.

The data used in this article are from Sina Weibo, China’s largest open social networking platform. Social media sites, such as Sina Weibo, YouTube, Twitter, and Facebook, may serve as vital conduits for companies to communicate with customers [25] and provide venues for individuals to communicate their perspectives, feelings, and experiences [26]. Sina Weibo is one of China’s largest and most prominent social media platforms. The number of monthly active Sina Weibo users rose to 584 million as of September 2022. This platform is ideal for studying the Chinese public’s interest in TGS. Using Sina Weibo data for research has been widely used in many fields. Pu et al. [27] collected posts on Sina Weibo about Japan’s nuclear wastewater discharge, used SnowNLP and LDA models to conduct sentiment analysis and topic extraction for user opinions, and explored the Chinese public’s emotions and opinions about the plan. Hu [28] used SnowNLP to calculate the emotional tendency of the “COVID-19 in China’s big cities” event on the Sina Weibo platform and analyzed the evolution of the microblog emotion of public health emergencies. Lu et al. [29] used SnowNLP to calculate the mood scores of Sina Weibo data in China’s heavily smoggy areas, providing a new perspective on the impact of haze on human psychology. Tang et al. [30] studied the subject development of Sina Weibo by using the LDA topic model to extract themes in various time periods. Wu et al. [31] collected data on the urban solid waste sorting policy on the Sina Weibo platform and conducted a sentiment analysis, which showed that nearly half of the public held a negative attitude towards the policy. Huang et al. [25] collected data on green consumption from Sina Weibo and analyzed the emotional tendencies of public opinions on green consumption. After collecting data on the Sina Weibo platform, this study adopted a text mining method to analyze public opinion of TGS. With the advancement of computer technology, we can now utilize text mining technology to collect useful data from vast amounts of text data. Text mining can efficiently analyze the unstructured data included in it, particularly with the advent of social media. Text mining technology is now used in a variety of industries, including express delivery services [32], finance [33], risk management [34], and sentiment analysis. Text mining allows practitioners or researchers to comprehend user requirements and investigate consumer ideas, feelings, and actions [35].

This research utilizes web crawler technology and text mining method to collect network data on Sina Weibo and studies the public’s attention and emotional inclination toward TGS. The SnowNLP module and Latent Dirichlet Allocation (LDA) topic model are used for sentiment and topic analyses. The main contributions of this study are as follows: 1. This paper discusses the application of Sina Weibo data in TGS research and provides a framework for text mining methods of corresponding data. 2. This paper explores the topics of positive and negative emotions of Chinese public attitude towards TGS and puts forward targeted solutions.

## 2. Materials and Methods

**Figure 1:**
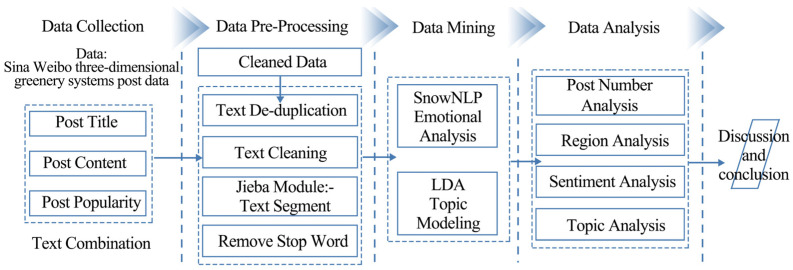
Research framework.

In this research, data were collected using web crawler technology; the unstructured data were analyzed using text mining technology; and the cleaned data were processed as a whole (Figure 1). The sentiment of TGS text data is then classified using the SnowNLP module. Finally, the LDA topic model is used to obtain the TGS topic content of the positive and negative emotions concerned by the public.

### 2.1. Data Acquisition and Preprocessing

The rapid expansion of the Internet has resulted in an abundance of network information, which has increased data accessibility. However, the huge growth of big data makes data collection problematic. Web crawler technology was developed to ensure that these data might be efficiently extracted and used. This technology is a program or script that automatically grabs web information according to certain rules. Furthermore, this technology includes deep web crawlers, focused web crawlers, incremental web crawlers, and general-purpose web crawlers [31]. In this study, a focused web crawler was used for data collection.

In this study, Python 3.10.6 was used to obtain data from the platform by using the following procedure:

- Simulate login. We simulated login using our Sina Weibo username and password (http://login.sina.com.cn/, accessed on 18 October 2022).
- Web search. This study used “three-dimensional greenery”, “building three-dimensional greenery”, “vertical greenery systems”, “green roof”, “wall greening”, and “balcony greening” as the keywords to obtain the original links from 28 August 2011 to 18 October 2022, based on the TGS and relative concepts.
- Data gathering. We obtained posting details, such as username, content, time of posting, forwarding number, number of likes, number of comments, and posting user information, such as authentication information and region.
- Data preservation. We saved the data in an Excel file that met the requirements.

The focus of this study is to use natural language processing technology to understand public attitudes toward TGS from social media data. The steps are as follows. During data pre-processing, we first eliminated duplicates. Texts with the same username, post content, and post year were considered duplicates, and only the first instance of the text was retained. Then, we eliminated text data with inconsistent formatting, missing information, or irrelevant material. We used the Naïve Bayes algorithm in the sklearn library to exclude text data with advertisements, missing information, and irrelevant material. Then, the text data are manually checked to meet the requirements. Finally, some unimportant information was removed from the text, such as special symbols, usernames, emojis, and site links in the reply or forwarding text.

This research used the Jieba Chinese word segmentation tool to realize the text preprocessing module. The list of stop words is an important factor affecting the segmentation results. This research integrated stop word lists from Baidu, the Harbin Institute of Technology, and the Machine Intelligence Laboratory at Sichuan University to accurately measure popular sentiment [25]. Finally, the list of stop words was continuously expanded according to the segmentation results, and some useless stop words were deleted until the segmentation effect met the requirements of the model.

### 2.2. Sentiment Analysis

We used the Snownlp module in the Python library for sentiment analysis. The Snownlp module itself comes with its own model, which can directly import data for sentiment analysis. Snownlp’s built-in model is focused on e-commerce, which is different from our research, so we trained a custom model on our own data. After the semantic analysis of the blog text, we randomly selected 500 positive emotion text and 500 negative emotion text as the training text. We import the sentiment tool to train a custom model sentiment file. We can then use the trained model to perform sentiment analysis on all blog posts. The sentiment value ranges from 0 to 1, representing both negative and positive possibilities. When the sentiment value is greater than 0.5, the sentiment tends to be positive. When the sentiment value is less than 0.5, the sentiment tends to be negative [27]. Python reads each line of text in the file through code, analyzes its sentiment and outputs the final result. This paper deals with the classification of data into two categories: positive (pos) and negative (neg). Its features, *w*_1_, *w*_2_, *w*_3_, …, *w_n_*, are independent of each other, and the formula is expressed as follows [36]:

(1) $$p\left(pos|w_{1},\ldots ,w_{n}\right)=p\left(w_{1},\ldots ,w_{n}|\;pos\;\right)\times \frac{p\left(pos\right)}{p\left(w_{1},\ldots ,w_{n}\right)}$$

(2) $$p\left(neg|w_{1},\ldots ,w_{n}\right)=p\left(w_{1},\ldots ,w_{n}|neg\right)\times \frac{p\left(neg\right)}{p\left(w_{1},\ldots ,w_{n}\right)}$$

(3) $$p\left(w_{1},\ldots ,w_{n}\right)=p\left(w_{1},\ldots ,w_{n}|pos\right)\times p\left(pos\right)+p\left(w_{1},\ldots ,w_{n}|\;neg\right)\times p\left(neg\right)$$

### 2.3. LDA Topic Model

LDA is an unsupervised probabilistic topic model, which is often used to model large-scale document collections. The basic idea is based on the assumption that when a user writes a document, he must have some definite topics in mind. After having the topics, he must select a word from the pool of words of a certain topic with a certain probability to explain the topic, and the whole document is equivalent to a mixture of different topics. The core idea of LDA is as in Equation (4).

*p* (words|documents) = ∑_topics_ *p* (words|topics) × *p* (topics|documents) (4)

We use topic strength to find hot topics. Topic strength describes the attention degree of a topic in a time window; that is, the more documents containing a topic in a time window, the higher the intensity of the topic. Assuming that $\theta_{x}^{d}$ is the proportion of subject *x* in document *d* and that $D_{t}$ is the set of texts on time window *t*, then the intensity of subject *x* on time window *t* is shown in Equation (5). After the topic intensity of all topics is calculated, the topic intensity threshold is set to obtain the topic with high attention. The calculation formula of topic intensity threshold is shown in Equation (6).

(5) $$\theta_{x}^{t}=\frac{\sum_{d=1}^{D^{t}}\theta_{x}^{d}}{D_{t}}$$

(6) $$T=\frac{\sum_{d}\sum_{x}\theta_{x}^{d}}{DX}$$

## 3. Results

We gathered 21,482 microblog entries regarding TGS and associated topics from 28 August 2011 to 18 October 2022. After the text was preprocessed, 13,978 posts were gathered in total.

### 3.1. Public Attention to TGS

The popularity of a post is measured by its total number of retweets, comments, and likes. The most popular posts were posted by bloggers with certain popularity, such as celebrities, shop exploration bloggers, information bloggers, and official accounts (Table 1). Table 2 shows the top ten users with the number of posts. The top three users are “Building green Association President unit—Heiner”, “Langting Garden”, and “Zhejiang Sol Garden”.

### 3.2. Post Number Analysis

**Figure 2:**
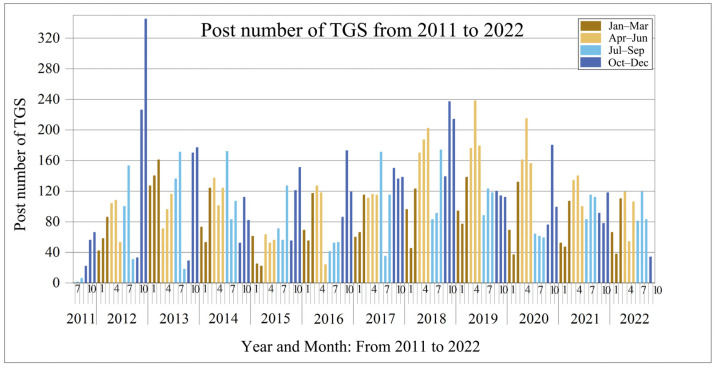
Post number of TGS from 2011 to 2022.

Posts about TGS on Sina Weibo were particularly few in 2011, as shown in Figure 2. The topic gained more attention starting in 2011, and the number of blog posts on the topic peaked by December 2012. The number of posts peaked from 2011 to 2012, increasing by 369%. On 18 November 2012, the Ministry of Housing and Urban-Rural Development, PRC issued the “Guidelines on Promoting the Healthy Development of Urban landscaping”, which clearly put forward the city residents travel “300 m to see the green, 500 m to see the garden” requirements and Additionally put forward the active expansion of green space, combined with municipal infrastructure actively develop wall, roof, balcony, bridge, bus station, parking lot and other 3D space green requirements. At the end of 2012, TGS workload was summarized across China, and TGS tasks were formulated during the 12th Five-Year Plan period. Shanghai is the first to take the lead. During the 12th Five-Year Plan period, Shanghai plans to build 1.5 million square meters of new TGS. In 2012, 30 TGS demonstration projects in Shanghai were publicized, among which Zhabei District of Shanghai built more than 10,000 square meters of green roof in 2012. This led to a surge in TGS posts at the end of 2012. Sina Weibo posts returned to rationality in 2013. The number of posts published has maintained a high level in the following years. This phenomenon shows that public attention to TGS continues after Sina Weibo has entered the adaptation stage. Another modest increase in the number of posts was observed between 2018 and 2019, followed by a return to normal after 2020. In terms of months, more blog posts were observed from March to June and from November to December, especially in November and December. Meanwhile, fewer posts were submitted between January and February. Most blogs were published in December, while the fewest were published in February (only 43.14% of the total number of blog posts in December). This result shows that the strong seasonal characteristics of TGS are also reflected in the number of TGS blog posts.

### 3.3. Region Analysis

**Figure 3:**
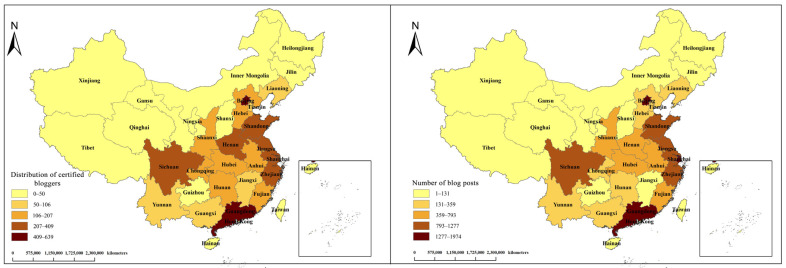
Regional distribution of verified users (**left**) and the number of posts (**right**).

In this research, we counted the provincial data of the users and obtained the regional distribution of the verified users of TGS (Figure 3 left) and the regional distribution of the blog posts of TGS (Figure 3 right). The legend in the lower left-hand corner shows the number of posts or verified users, with darker colors indicating higher numbers of posts or verified users in the region. Overall, a minimal change is observed between the two figures. Users in western and northeastern China pay less attention to TGS compared with the other Chinese areas, while Beijing has the highest attention to TGS. Meanwhile, users in Beijing and Guangdong showed more interest in TGS compared with other provinces, followed by Sichuan Province, Shandong Province, Jiangsu Province, Shanghai, and Zhejiang Province. Users in coastal areas and areas with warm climates and developed economies pay more attention to TGS.

### 3.4. Sentiment Analysis

**Figure 4:**
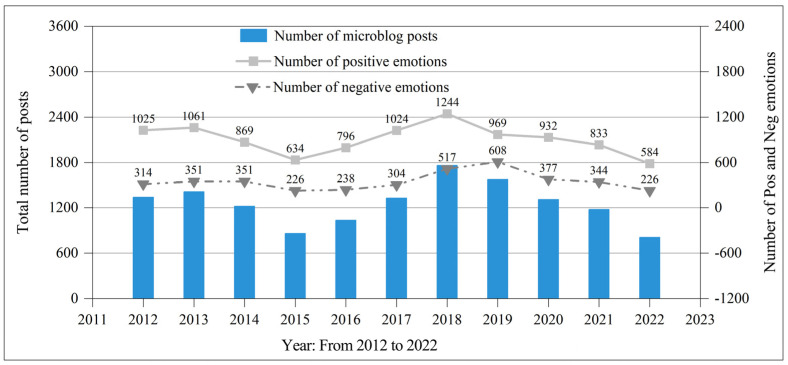
Number of positive and negative emotions and total number of posts in TGS during 2012−2022.

We conducted emotional classification of TGS data on Sina Weibo by using the SnowNLP module. The results show that Chinese people have a favorable attitude toward TGS (positive posts/total posts: 10,092/13,978, 72.20%). From the perspective of time evolution, the positive posts from 2012 to 2022 accounted for 76.55%, 75.14%, 71.23%, 73.72%, 76.98%, 77.11%, 70.64%, 61.45%, 71.20%, 70.77%, and 72.10%, all stable at approximately 75%. Positive emotions are dominant, and the fluctuation value is small. Given that the information on blog posts in 2011 only covers the period from August to December, the data volume is small, which affects the overall change trend of the number of blog posts. Accordingly, the 2011 data are deleted in Figure 4. Figure 4 depicts the amount of Weibo posts about TGS by Chinese residents over the last decade. After 2015, the number of TGS posts had a generally constant growth pattern until 2018, when it peaked and then returned to normal. The maximum number of microblog posts of TGS every year was 951 more than the lowest. When formulating China’s 13th Five-Year Plan in 2015, the government proposed the elevation of ecological civilization construction as a national strategy, the strengthening of comprehensive environmental management, the improvement of the level of ecological civilization, and the promotion of green development [37]. The government also proposed to strengthen the construction of sponge-shaped buildings and residential areas, sponge-shaped roads and squares, and sponge-shaped parks and green spaces. At the 19th National Congress of the Communist Party of China held at the end of 2017, the state advocated for the peaceful coexistence of man and nature [38]. The 19th National Congress urged engagement in global environmental governance, implementation of emission reduction commitments, and contribution to global ecological security. Additionally, the 19th National Congress advocated programs to build green families, schools, communities, and travel. The abovementioned policies have greatly increased citizens’ attention to TGS.

After 2018, the number of TGS posts decreased year by year. From 2018 to 2019, the number of positive posts about TGS significantly decreased, while the number of negative posts increased. In 2019, positive and negative posts accounted for 61.45% and 38.55%, respectively. The public’s health awareness and health concerns have dramatically risen due to the outbreak of COVID-19 in 2019 and 2020, and the public’s online attention to health care accounts for a large proportion [39]. In addition, many places in China have implemented quarantine status due to COVID-19 [40]. The spread of COVID-19 has caused a considerable number of problems in the construction industry [41], such as delaying and stopping projects that were already in progress before the pandemic [42]. The construction industry has been affected by COVID-19 to varying degrees [43]. These effects can be divided into effects on the construction site (for example, worker safety and health) and off-site effects (for example, contractual consequences in the context of COVID-19) [44]. Wang et al. [45] highlighted various risk factors that create obstacles for building projects due to COVID-19 as follows: accessibility of locations, availability of labor, lack of building supplies, and limited COVID-19 health protective equipment. The abovementioned reasons also led to a decrease in the construction of TGS of buildings, and the posts on Sina Weibo about TGS of buildings also decreased year by year. Next, LDA topic modeling and analysis of the positive and negative sentiment data will be conducted to further elucidate public concern around TGS.

### 3.5. Topic Analysis

**Figure 5:**
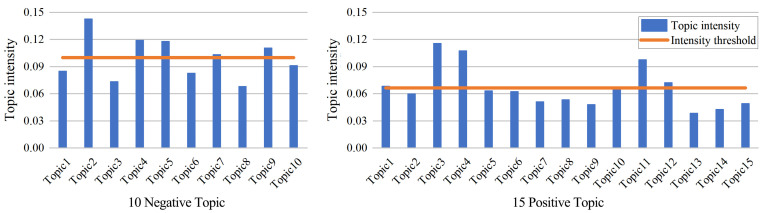
Distribution of topic intensity values.

First, the LDA model was utilized to summarize the positive and negative emotion text data of TGS. To guarantee the topic’s independence, we find the ideal number of topics by combining the perplexity curve with the LDAvis visualization system. Based on the perplexity calculation [46], the optimal topic number of positive emotions was 15, and the optimal topic number of negative emotions was 10. In order to find hot topics, we calculated the topic intensity of each topic and the intensity threshold. Figure 5 shows the distribution of topic intensity of positive and negative emotional text topics. The orange horizontal line represents the intensity threshold, and the vertical axis represents the topic intensity of each topic. When the topic intensity value is greater than the intensity threshold, the topic is a hot topic. The hot topic numbers among the 15 positive topics are 1, 3, 4, 10, 11, and 12. The hot topic numbers among the 10 negative topics are 2, 4, 5, 7, and 9. We use pyLDAvis to obtain the visual chart of positive and negative emotional text. The LDAvis visualization diagram can be mainly divided into left and right parts. The dot on the left represents the topic, and the dot size is determined by the document frequency corresponding to the topic [25]. The right portion of the diagram depicts are the keywords corresponding to the topic, sorted by generation probability.

#### 3.5.1. Positive Emotions for TGS Based on the LDA Model

**Figure 6:**
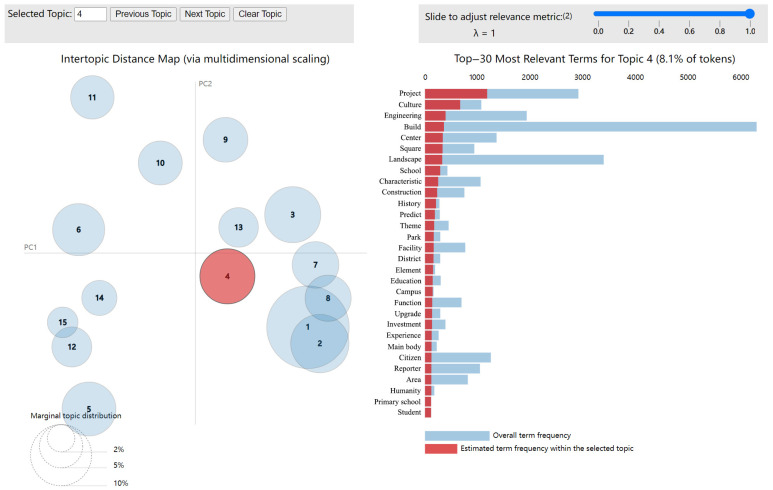
Topic 4 of topic models (positive sentiment) [51,52].

The visual chart of the positive sentiment text obtained using pyLDAvis is shown in Figure 6. The positive sentiment text topics and their top 30 keywords are presented in Table 3. We will mainly explain popular topics. In Topic 1, “park”, “green space”, “greenway”, “garden”, and “park green space” are high-frequency words, indicating that TGS has been integrated into the construction of parks. The public finds that TGS is integrated into many types of parks, such as integrated parks, strip parks, and pocket parks. The park has increased into the 3D flower beds, roof greening, and wall greening. At present, there are various forms of TGS, and residents say these green projects make them more happy. The facade of many public toilets in parks is laid with vertical greenery to blend in with the surrounding environment. The integration of TGS into park construction is not only an inevitable means to increase urban greening rate, but also the result of government policy guidance and people’s yearning for green natural life.

The central idea of Topic 3 is rural revitalization and rural landscape construction. Words such as “ecology”, “rural”, “village”, “tourism”, “economy”, and “agriculture” were used more frequently. TGS has been deeply integrated into rural revitalization, rural landscape construction, and improvement of the living environment. Since the New Countryside construction campaign, the central and local governments have increased their investment in rural roads, water conservancy, improvement of the living environment, and construction of beautiful and livable villages. Rural landscape construction began to promote TGS, which is in line with the general direction of the country. Under the TGS color landscape decoration, some rural areas began to vigorously develop rural tourism. At the same time, rural agriculture and the economy also developed in tandem.

China has started to increase young people’s knowledge of environmental preservation, as shown by Topic 4. Keywords such as “school”, “education”, “campus”, “humanity”, “primary school”, and “student” can be seen. Chinese education has integrated the contents related to TGS and low-carbon life into the construction of schools to ensure that they may comprehend the essence of TGS and environmental conservation as future energy consumers. In this approach, people may be better directed to practice a low-carbon life to achieve the goal of peak carbon dioxide emissions and carbon neutrality in China earlier. In the past decade, many primary and secondary schools and colleges in China have implemented TGS projects. Schools have increased campus greenery by VGS, hanging gardens, and green libraries and actively organized the creation of “low-carbon schools” and “green schools”. The creation of a “low-carbon school” aims to start with children, bring the concept of low carbon into schools, cultivate children’s concept of green and low carbon, care for the environment, develop a low-carbon lifestyle, enhance low-carbon education, and create a low-carbon atmosphere.

In Topic 10, “heat island effect”, “temperature”, and “ecology” are high-frequency terms, showing that urban pollution and urban high temperature environment are of great concern to inhabitants. In hot areas of some Chinese cities, the summer heat has continued to exceed 40 °C in recent years, while long-time outdoor workers (such as soldiers, firefighters, military officers, athletes, and construction workers) have remained at their jobs for various reasons. This phenomenon has led to a steady increase in heat stroke, which has a high mortality rate and irreversible damage to the human body [47]. Studies by the United Nations Environment Programme indicate that the building sector, which accounts for 38% of the total world energy-related CO_2_ emissions, will play a significant role in reaching our objective of limiting global warming to well below 2 °C [48]. Besir et al. [49] asserted that green roofs and outside walls are important strategies for decreasing greenhouse gas emissions and building-related energy consumption. Karteris et al. [50] revealed that the installation of a green roof may lower the amount of heat entering a building by approximately 80% during the summer. The public also recognizes that TGS can bring huge environmental and economic benefits to cities by alleviating the UHI effect, improving building energy efficiency, reducing the load of urban rainwater treatment systems, and enriching urban species diversity. TGS is also an important way to beautify urban landscapes, improve the climate environment, and enhance ecological benefits. The keywords “business”, “hotel”, “residence”, and “office” show that TGS has been integrated into commercial buildings, public buildings, and individual residences. Some citizens mentioned that the work efficiency in offices with VGS has been greatly improved. Furthermore, VGS has been added to the exterior walls of public toilets in many cities, giving visitors profound green visual enjoyment.

Topic 11 mainly stands for green building construction, in which “architecture”, “green building”, “roof garden”, “architectural design”, “designer”, “sustainable”, and “ecological building” are the key words. Green building refers to a building that saves resources to the greatest extent, protects the environment, reduces pollution, and provides people with healthy, comfortable, and efficient use of space within the whole life cycle of the building. Green building can also be called sustainable development building, ecological building, return to nature building, and energy saving and environmental protection building. TGS, as an increasingly popular form of greening, plays an important role in green building construction.

The central idea of Topic 12 is the application form of TGS. Words such as “roof”, “garden”, “balcony”, “wall”, “green clothes”, “landscape painting”, and “color painting” are frequently used. The types of TGS include green roof, wall greening, bridge greening, balcony greening, and corridor greening. TGS is also applied in a variety of forms. For example, the roofs and walls of public houses are decorated with evergreen plants such as *Chlorophytum* and *Rohdea Roth*, and graffiti and color painting such as locomotive and panda are painted on the front of the house. Such costumes bring the public a beautiful scenery line. Lush greenery adds a lot of color to the building, and this way of greening makes the public feel very creative.

#### 3.5.2. Negative Emotions for TGS Based on the LDA Model

**Figure 7:**
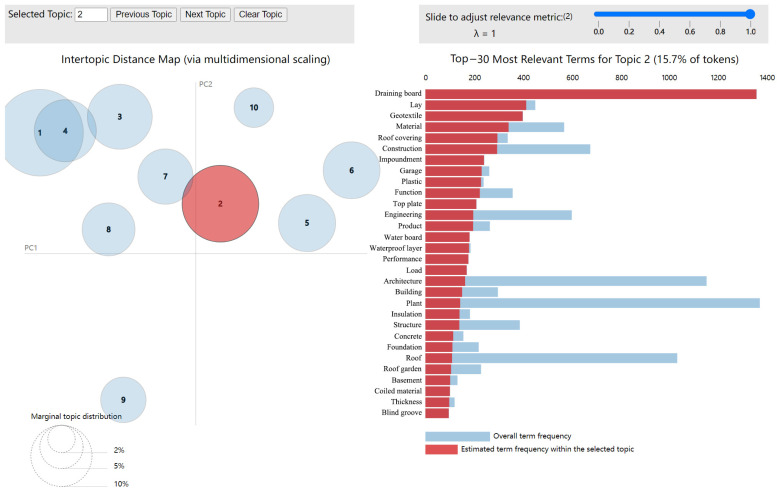
Topic 2 of topic models (negative sentiment) [51,52].

The visual chart of the negative sentiment text obtained using pyLDAvis is shown in Figure 7. The negative sentiment text topics and their top 30 keywords are presented in Table 4. We will mainly explain popular topics. According to the analysis of negative emotion text data, many Sina Weibo users posted their opinions from the perspective of the building itself, the indoor environment, plant care, and their own conditions. In Topic 2, “geotextile”, “waterproof layer”, “load”, and “structure” are widely discussed. The public expressed concerns about the load-bearing, waterproofing, guardrail, wall cracks, and plant firmness of the building structure. They believed that increasing the TGS of the building would damage its original structure and increase its danger factor. The collapse of the green roof at the Sports Center of City University of Hong Kong in 2016 reminds people that green roofs must be planned, designed, and evaluated by professional organizations. In June 2017, the Nanyang Urban Management Bureau invited Ma Liya and Han Lili, two experts from the Beijing Roof Greening Association, to explain on site. Experts have noted that green roofs must consider safety issues, load safety, structure safety, waterproofing safety, and plant growth safety. Moreover, experts have noted that different houses have different bearing capacities, and green roofs must be the first load assessment of the building. This notion is consistent with Cascone’s study that the first step in retrofitting buildings with a green roof is to assess the current load-bearing capability [53]. Cascone et al. [53] showed that the maximum allowable additional load for installing green roofs on existing buildings is 1.46 kN/m^2^.

The words “Management”, “inspection”, “municipal”, and “unit” in Topic 4 are all about the municipal construction management of TGS. In some old residential areas, roof greening and wall greening have safety risks, aging facilities and equipment, damage, and other problems. This not only affects the green appearance but also causes damage to the building structure. In order to further improve the living environment, we should accelerate the comprehensive environmental improvement of the old residential areas. In severe weather conditions such as typhoons, snowfall, and continued high temperatures, the public has suggested conducting inspections on epidemic prevention and control, fire safety hazards, air pollution prevention and control, cold and freezing prevention of plants, drought resistance and green protection of plants, and daily work safety.

The words “plant”, “growth”, “technology”, and “variety” in Topic 5 are all about the planting and subsequent maintenance of TGS plants. The majority of people are concerned about the subsequent maintenance and watering of plants, whether plants are easy to care for, and whether they have automatic watering devices. Balcony greening plants that take a long time to grow or whose roots cannot reach out or climb up to the roof are also a public concern. The firmness problem of plants, such as wind and rain and other weather, is related to the safety of residents.

Topic 7 shows the negative public sentiment towards TGS architectural design. “Architecture”, “roof”, “green building”, and “architectural design” are key words. The public pointed out that the difficulty of the implementation of TGS lies in the difficulty of promotion, construction, and maintenance. The spatial layout of TGS is restricted by the inherent plan of the building and the bearing capacity of the building structure. Compared with the open garden, TGS design is not only complex but also related to the architectural design, architectural structure, and hydropower and other types of work cooperation.

In Topic 9, “Owner”, “Property”, “roof”, “fire escape”, “Water leakage”, “vegetable growing”, and “Property management” are all about the property management aspects of TGS House. Some citizens said that after the residential area was handed over, the previous publicity of TGS did not materialize. Property company costs are high, but the service is not in place. The owner planted vegetables on the roof, connected water and electricity privately, and watered flowers with water from fire pipes. Other neighbors worry about safety risks, and some property companies cannot adjust the matter. Private planting of vegetables on the roof belongs to the regular roof greening or illegal construction projects need to be examined. Roof planting vegetables need to pay attention so that the floor bearing, roof waterproof layer, and insulation layer are not destroyed, otherwise the roof will appear to have a water leakage phenomenon.

Some people indicated that the TGS of buildings will affect the indoor environment of houses; plants will increase mosquitoes; and they are afraid to open windows in summer. This phenomenon is the same as Liu et al.’s argument that the public will consider the rise of mosquitoes, snakes, insects, and ants produced by the increase in vegetation when faced with vertical greening houses [54]. Moreover, some residents indicated that TGS affects indoor lighting, and lights need to be turned on during the day. TGS also affects drying clothes, and indoors will become damp. Residents in the coastal areas of Guangdong asserted that high-rise buildings with VGS can be dangerous in typhoon weather.

## 4. Discussion

The abovementioned research explored the concerns and emotional orientation of the Chinese public on TGS based on the Sina Weibo platform. The public’s concern for TGS has increased in thought and behavior over time. Most people have a positive attitude about TGS, believing that it can improve the urban ecological environment. However, 27.8% of the people still have negative feelings toward TGS from many aspects.

### 4.1. Users Pay Varying Attention to TGS Due to the Different Geographical Locations and Account Authentications of Users

First, users with verified microblogs, complete information, and high influence pay more attention to TGS, which can increase account fans’ attention to TGS. Second, the public in economically developed areas, warm climate areas, and coastal areas pays more attention to TGS. In major Chinese cities, such as Beijing and Shanghai, the government is paying more attention to the formulation and implementation of TGS policies. Finally, Sina Weibo users include certified and noncertified users. Certified users include organization and individual certified users. The identity of the institutionally certified users obtained by the TGS search can be divided into government agencies, research institutions, consulting institutions, development units, design units, suppliers, construction units, magazines, and media. Personal certified users are mainly related personnel of the above units, fashionistas, information bloggers, shop exploration bloggers, and famous home bloggers. The outcome indicates that the development of TGS needs the efforts of all parties in the country. National governments need to develop appropriate policies and guide enterprises, construction units, and development units to publicize and apply TGS. When citizens pay attention to and perceive the increase in TGS in commercial and public buildings, the public will have an emotional preference for TGS in buildings and behavioral intention. When promoting TGS, star and celebrity effects can be used to accelerate its publicity and promotion.

### 4.2. Policy Plays an Essential Role in Guiding the Public’s Selection and Use of TGS

Based on the time and spatial aspects of the public’s emotional orientation toward TGS, the policy plays a crucial role. In 2016, 178 parties from all around the globe signed the Paris Agreement and vowed to attempt to limit global warming to 1.5 °C [55]. The Communist Party of China has been exerting continuous efforts to solve global ecological problems, from actively promoting the United Nations Framework Convention on Climate Change in 1992 [56] to Comrade Xi Jinping’s attendance at COP21 in 2015 and signing the Paris Agreement. At the 2016 G20 Hangzhou summit, China and other nations pledged to push the Paris Agreement’s entrance into effect as quickly as possible. The report of the 20th National Congress of the Communist Party of China indicated to “advance the establishment of a natural reserve system with national parks as its core” and “actively and steadily advance peak carbon dioxide emissions and carbon neutrality”. China has made unique contributions to the solution of global ecological problems. Meanwhile, the Chinese government has enacted a huge number of TGS and green building policies, such as promoting the development of TGS, technical standards for the application of TGS in buildings, and national green building evaluation standards, which have gradually come into people’s view [57]. However, the implementation time of TGS in China is relatively late compared with the development of foreign TGS policies, and legal support from the national to the local levels is inadequate. Only the words “advocating TGS” were proposed at the national level. Local regulations on TGS are mainly technical standards at the specific implementation level, which have a limited effect on the promotion of TGS. According to the local characteristics, some cities make explicit the specific requirements of TGS construction projects under their jurisdiction, which has a certain mandatory role. However, the mandatory requirements of various cities lack a legal basis and unified standards.

### 4.3. The Public’s Emotional Topics of TGS Are Different

In this work, sentiment analysis and topic modeling contribute to the analysis of different emotional topics of the public toward TGS. We should pay attention to the negative emotions related to the price, time cost, safety issues, and policies of TGS houses. The implementation of TGS is hampered by the price of TGS houses, the follow-up maintenance of plants, the increase in indoor mosquitoes, and the lack of mandatory policies. This finding is not surprising because some members of the public have negative emotions toward TGS due to their lack of understanding of it. In addition to meeting the eight safety requirements of housing load-bearing, structural drainage, anti-leakage, anti-typhoon, fire protection, heat insulation, plants, and insects, TGS should also consider soil safety, material safety, no contaminated soil, no chemical plastic products, etc. By contrast, public education, a good indoor and outdoor TGS environment, and purified urban air can stimulate the public’s positive mood toward TGS. The continuous promotion of the TGS movement by the Chinese government can improve the public’s attention to it. When the public realizes that TGS can improve the urban ecological environment and their own health, they will actively support it. At exhibitions, such as the 2019 Beijing International Horticultural Exposition and the 2021 China Shanghai Three-dimensional Greening Exhibition, major platforms have opened special TGS exhibitions, which is a new measure to promote TGS. In the future, we can develop prefabricated greening with public participation that can be combined with homes, such as 3D farms that combine TGS and ecological farms, and truly realize regional circular agriculture with zero waste emissions. In addition, high-rise TGS with natural succession and less human participation can be developed to improve the public’s positive emotions toward TGS. To actively promote the construction of TGS clusters or the renovation of existing buildings, it can be combined with the artificial intelligence remote system [1] to enhance the development scale of TGS in China.

## 5. Conclusions

Using social media platforms to study the public’s focus and emotional views on TGS can help researchers formulate government policies and determine research directions. TGS is important in the development of urban ecological environments. In this study, the SnowNLP model was used to classify the emotions of the text data of TGS, and the LDA topic model was utilized to analyze the topic focus of the positive and negative emotion data to explore the attitudes and driving factors of the Chinese public toward TGS. The survey results show that most of the public has a positive attitude toward TGS, and economically developed areas pay more attention to TGS. Different regions in China pay varying attention to TGS: eastern region > central region > western region > northeast China. The Chinese public’s attention to TGS has significantly increased with the change in the government’s governance concept, but it still needs to be improved. The enhancement of teenagers’ awareness of environmental protection, the completed TGS landscape, and mandatory and incentive policies are conducive to the implementation of TGS. When the public is faced with high prices of TGS housing, follow-up maintenance of plants, safety problems in dangerous weather, increased indoor mosquitoes, structural damage caused by TGS buildings, water leakage, and other problems, they easily produce negative emotions about TGS housing.

We can summarize some suggestions based on the results of the data analysis. First, policymakers should gradually create a sound TGS policy framework system to ensure the comprehensive development of mandatory, incentive, and aid policies from the national to the local levels. The legalization and standardized development of TGS policies must be promoted, and the integrity and effectiveness of the TGS policy system from top to bottom must be consolidated to ensure that different levels and stages of policy implementation have a concrete basis to follow. We should further increase support for TGS development in the central and western regions and Northeast China to narrow the regional gap. Policy formulation also needs to consider the details of education, family, work, and so on to strengthen the implementation of policies. Second, social media platforms should be used to promote and encourage public participation. Star and celebrity effects must be used to publicize TGS, strengthen media professional marketing, and expand the influence. Public awareness and interest in the multiple uses of TGS and knowledge of plant maintenance should be promoted through marketing and educational programmers. Citizen participation in management and decision-making processes should be encouraged. Relevant organizations and associations must also be encouraged to frequently host exhibits on the topic of TGS to provide the public with an opportunity to learn about TGS. Third, the subsequent maintenance of TGS construction must be ensured. TGS should mainly use plants with strong adaptability, drought tolerance, and low maintenance, such as *Sedum* [58]. These plants typically do not need a separate irrigation system because they rely only on natural precipitation, which can solve the problem of plant care. Plant species that can thrive in the local climate should be selected for TGS. The construction side should ensure the service life of TGS to keep plants from wilting after a year or two. Furthermore, developers and property management units should check the quality of TGS housing according to the stipulated time to avoid imperfect supporting facilities, roof leakage, wall cracking, and other serious problems. New technologies, such as automatic drip irrigation, energy savings, and water savings, can be used for the daily management of TGS, further improving the fine management level of TGS and providing the public with a new life experience.

This article has some limitations. First, Sina Weibo software has some restrictions on searches, so the data may not be comprehensive. Second, Sina Weibo users may not cover all age groups, such as the elderly and children. Finally, this work does not use other national social software to investigate the TGS situation of other countries, such as Twitter and Facebook. After that, we will mainly focus on these questions in further studies.

## Figures and Tables

**Table 1:** Ten most popular posts’ essential information.

No.	Account Authentication	Gender	Post Time	Post Hotness
1	Fashionista	Male	24 March 2022	8215
2	Environmental protection official account	Male	14 November 2019	2649
3	Food blogger	Male	19 November 2017	2304
4	Famous home blogger	Male	27 March 2021	1868
5	Shop exploration blogger	Female	21 October 2021	1278
6	Official news account	Female	30 June 2021	1089
7	Chongqing local new power blogger	Male	28 June 2019	1054
8	Chengdu local information blogger	Male	24 November 2018	1038
9	Legal person account of news network	Male	23 November 2018	870
10	Official account of the Park Bureau	Male	26 September 2019	813

**Table 2:** Basic information about the top 10 authenticated users with the highest number of microblog posts.

No.	Authenticate Account Name	Post Number
1	Building Green Association President Unit—Heiner	407
2	Langting Garden	314
3	Zhejiang Sol Garden	237
4	Chinese Journal of Building Waterproofing	195
5	Shi Linong Lao Liao	169
6	World Roof Greening Association	165
7	Easy Green Valley Roof Greening	139
8	China Building Greening Network	117
9	Huantong International Development	96
10	Green Shanghai	84

**Table 3:** Fifteen positive topics and keywords.

No. Topic	Topic Explanation	Topic Keywords
Topic 1	Construction of park	Park, construction, green space and urban area, landscape, ecology, roads, green road, garden, the green, citizens, engineering, center, work, landscape architecture, park green space, landscape, pocket, project, characteristic, program, whole city, start, square, journalists, avenue, focus, planning, quality, small garden
Topic 2	Urban and rural environmental construction	Work, renovation, roads, management, garbage, streets, facilities, and life, construction, the crowd, focus, engineering, village, comprehensive, urban area, wall, city appearance, city, classification, traffic, special, refinement, quality, specification, settings, departments, alley, standard, parking lot
Topic 3	Rural revitalization and rural landscape construction	Ecology, forest, construction, development, country, afforestation, countries, industries, village, focus, green, tourism, construction, planning, resources, culture, economy, patterns, human settlements, forestry, rural, characteristic, agriculture, strategy, tree species, the forest, the city, system barrier, road
Topic 4	Campus and education	Project, culture, engineering, construction, center, square, landscape, school, the features, construction, history, estimates, theme, park, facilities, site, elements, education, campus, functionality, upgrading, investment, experience, main body, citizens, journalists, area, the humanities, primary school, students
Topic 5	TGS plant application form	Landscape, plants, flowers, flower pots, garden, path, stereo, fresh flowers, modeling, avenue, green wall, varieties, characteristics, flower boxes, balcony, tracery wall, decorate, park bureau, vines, square, guardrail, citizens, theme, color, green, hefei, journalists, construction, lawn, garden
Topic 6	Plant ecological benefit	Plants, roof, air, buildings, green, decoration, utilization, purification, materials, ecology, growth, living, urban greening, wall surface, cooling, temperature, ground, technology, three-dimensional, land, landscape, noise, garden, roof garden, development, desktop, urban heat Island effect, regulation, wall, function
Topic 7	Community participation	Community, activities, work, construction, residents, units, plant trees, ecology, green, landscaping, national, social, community, service, duty, streets, propaganda, department, life, school, jurisdiction, start, hospital, whole country, management, their homes, citizens, public welfare, development, trees
Topic 8	Policies and Regulations	Construction, project, unit, world, wall, urban greening, urban three-dimensional greening, management, guidelines, standards, planning, public buildings, subsidies, land, methods, countries, housing, journalists, work, opinions, landscaping, green rate, regulations, government, micro magazine, residential area, policy, indicators, building energy efficiency, green space
Topic 9	Construction technique	Technology, development, science and technology, enterprise, company, green, company limited, construction, industry, engineering, products, garden, international, professional, green building, whole country, service, expert, gardening, production, system, standard, energy, materials, industry, construction, field, center, forum, and ecology
Topic 10	Environmental governance benefit	Project, green, and international business, research, center, life, hotel, residential, office, grow vegetables, garden, heat island effect, the balcony, platform, planning, company limited, building area, waste gas, the temperature, experience, area, land, buildings, group, afforestation, ecological, headquarters, idea, high-end
Topic 11	green building construction	Architecture, green building, green, garden, development, roof, concept, roof garden, utilization, architectural design, home, building, designer, condition, sustainability, meaning, technology, full use, representation, eco-architecture, solar energy, height, exterior wall, vegetation, ecology, architect, whole, office, idea, function
Topic 12	TGS plant application form	Roof, garden, balcony, metope, plants, village, forest, citizens, wall, landscape, journalists, stereo, green, rose, tianfu, plane, ecology, house, scene, the green, slope protection, household, landscape painting, originally, utilization, tunnel, projects, colored drawing or pattern, characteristic, vegetable garden
Topic 13	Sponge city	Sponge construction, city, rain, system, landscape, ecology, using, sponge, project, idea, facilities, green land, planning, measures, irrigation, technology, square, settings, rendering, park, area, road, garden, device, circulation, sewage, function, runoff, purification, community
Topic 14	Elevated TGS design	Ivy, green, courtyard, villa, waterfall, overpass, second ring road, bee, garden, library, pier, bridge, wall, viaduct, roof garden, wire bridge, construction, Earth, university, family, global, contribution, transportation, high technology, education, bridge pillar, food, pedestrian bridge, exhaust, planting
Topic 15	TGS design for public buildings	Public toilet, case, consultation, landscape design, landscape, building greening, vertical green wall, plants, high-rise, company, customer service, toilet, intelligent, system, roof garden, free, automatic, exterior wall, appearance, aesthetics, hotline, interior design, wall, roof, training, drainage board, green plant, tea tree, postgraduate entrance examination, plastic

**Table 4:** Ten negative topics and keywords.

No. Topic	Topic Explanation	Topic Keywords
Topic 1	Residential area management	Village, residents, community, wall, streets, garbage, work, roads, construction, infrastructure, life, journalists, advertising, green belts, jurisdiction, area, mass, unit, the activity, urban area, villagers, houses, residential, citizens, pavement, engineering, courtyard, management, environmental health
Topic 2	Building structure	Drainage board, laying, geotextile, material, roof, construction, water storage, garage, plastic, Function, roof, engineering, Product, water board, waterproof layer, performance, load, building, building, plant, insulation, structure, concrete, foundation, roof, roof garden, basement, coil, thickness, blind ditch
Topic 3	Construction project	Project, engineering, construction, wall, landscape, culture, development, flower boxes, architecture, system, ecology, roads, construction, as a whole, style, reporter, square, tourism, tourists, modeling, time, characteristic, citizens, toilets, pipes, foundation, company Limited, expect, wall, business
Topic 4	municipal construction	Work, management, business, service, alley, facilities, inspection, activity, situation, roads, epidemic, construction, waste, personnel, department, construction site, green space, vehicles, municipal, masses, unit, the control and prevention, measures, operation, business, equipment, comprehensive, build, platform, area
Topic 5	Plant planting and subsequent maintenance	Plants, roof, ecology, growth, roof, technology, formaldehyde, substrate, temperature, green, grass, air, methods, soil, flower pots, *Sedum*, cooling, utilization, rainwater, condition, variety, development, pollution, influence, climate, air conditioning, high temperature, ground, land, ability
Topic 6	Plant species	Plants, growth, cultivation, materials, Boston ivy, flower, garden, potted plants, production, flowering, vines, flowers, shrubs, seeds, *Euphorbia humifusa* Willd, gardens, origin, cover, lianas, woody, bonsai, leaves, soil, white, scaffolding, region, scientific name, *Pharbitis*, herbaceous, oval
Topic 7	Architectural design	Architecture, green, technology, roof, world, Green building, house, roof Greening Association, structure, living room, center, place, architectural design, micro journal, renderings, international, project, open, commercial, living, restaurant, landscape, hotel, kitchen, industry, wall, villa, exterior wall, old house, house
Topic 8	TGS housing purchase	Residential, Christmas tree, community, elevator, house, standard, high-rise, structure, house, floor, housing, living area, layout, building area, factor, developers, buyers, artificial lawn, buy a house, decorations, price, planning, green rate, apartment type, sunshine, real estate, life, high-rise residential, distance, quality
Topic 9	TGS housing property management	Owner, property, information, company, developer, experience, drawings, roof, ninemsn, gifts, fire access, water leakage, home, vegetables, house, reporter, community, company limited, I will, publicity, fire, service, property management, vegetable garden, economy, property company, opinion, university, official, group
Topic 10	TGS design	Garden, balcony, lawn, plants, articles, case studies, headlines, office, roof garden, planting grass, the top floor, wall vertical greening, landscape, designers, glass, experts, gardening, professional, decoration, friends, life, apartment, artificial, projects, pictures, sod, rights protection, the green wall, courtyard, room

## Data Availability

Not applicable.
